# Oversight in Surgical Innovation: A Response to Ethical Challenges

**DOI:** 10.1007/s00268-018-4565-2

**Published:** 2018-03-13

**Authors:** Saksham Gupta, Ivo S. Muskens, Luis Bradley Fandino, Alexander F. C. Hulsbergen, Marike L. D. Broekman

**Affiliations:** 1 Harvard Medical School, Boston, MA USA; 2 Department of Neurosurgery, Brain Center Rudolf Magnus, University Medical Center Utrecht, HP G03.124, PO Box 85500, 3508 GA Utrecht, The Netherlands

## Abstract

**Background:**

Surgical innovation has advanced outcomes in the field, but carries inherent risk for surgeons and patients alike. Oversight mechanisms exist to support surgeon-innovators through difficulties associated with the innovation process.

**Methods:**

A literature review of ethical risks and oversight mechanisms was conducted.

**Results:**

Oversight mechanisms range from the historical concept of surgical exceptionalism to departmental, hospital, and centralized committees. These fragmentary and non-standardized oversight mechanisms leave surgeon-innovators and patients open to significant risk of breaching the ethical principles at the core of surgical practice. A systematized approach that mitigates these risks while maintaining the independence and dignity of the surgical profession is necessary. We propose an oversight framework that incorporates multiple structures tailored toward the ethical risk introduced by different forms of innovation.

**Discussion:**

We summarize ethical risks and current regulatory structures, and we then use these findings to outline an oversight framework that may be applied to surgical practice.

## Introduction

The drive to innovate has resulted in significant improvements in surgical outcomes. Surgical innovation occurs in contexts ranging from individual cases with unique anatomic features to clinical trials, though there is no single, universal definition of surgical innovation. Consequently, surgical innovation can present a challenge by blurring the distinction between experimentation and clinical care. The Belmont Report defines innovative care as “practice that departs significantly from the standard or accepted” and posits that innovative care that deviates significantly from the norm should be formally researched with oversight in place [1, 2].

The distinction of research and clinical motivation rests on their respective motivation: the primary goals of operative innovation in the clinical and research contexts, respectively, are beneficence to optimize patient care and experimental evaluation to generate generalizable knowledge. Experimental techniques intended to test the new technique with equipoise fall into the research category that receives oversight from institutional review boards (IRBs). However, surgical innovation currently falls outside the realm of oversight since it is often intended to benefit an individual patient rather than systematically investigate a procedure. This type of innovation is exemplified by the hypothetical case of an ostomy between the common bile duct (CBD) and hepatopancreatic ampulla to prevent malabsorption for an infant born with type I biliary atresia with preserved proximal CBD.

The current lack of consensus on oversight mechanisms for procedural innovation leaves surgeons and patients vulnerable to significant risk which carries ethical implications for surgical practice [3]. No standardized approach exists to aid surgeons in evaluating the ethical challenges inherent in surgical innovation. This perspective focuses on the ethical challenges associated with surgical innovation and proposes an oversight framework to regulate it.

## Mechanisms for oversight

Various methods to oversee operative innovation have been suggested, ranging from regulation by the operator alone (surgical exceptionalism) to formal evaluation and oversight for every innovation (Table 1) [2, 4]. This range of opinions highlights the delicate ethical balance between assuring patient safety without stifling innovation.

**Table 1:** Summary of oversight mechanisms

Oversight level	Benefits	Drawbacks
Surgical Exceptionalism	Surgeon knows patient best, professional dignity and autonomy maintained, expedient	Susceptible to individual biases and COIs, interoperator inconsistencies, no support for surgeons
Departmental	Surgeon knows patient best, multiple opinions incorporated, professional dignity and autonomy maintained, expedient	Susceptible to institutional biases and COIs, interhospital inconsistencies
Institutional	Multidisciplinary opinions incorporated, surgeon protected by legal and ethical expertise	Interhospital variability, professional independence may be compromised, moderately costly and time-intensive
Regional/national	Multidisciplinary opinions incorporated, sets precedents for entire field, no interoperator and interhospital variability	Subject to biases of the field, highly costly and time-intensive, assessment by evaluators removed from patient
IRB	Multidisciplinary opinions incorporated, protocolized, standardized, transparent	Moderately costly and time-intensive, assessment by evaluators removed from patient

### Surgical exceptionalism

Surgical exceptionalism is characterized by regulation of an innovation by the surgeon performing the procedure without formal oversight [4]. Some argue that features unique to the surgical profession—difficulty in measuring surgical technique, reproducing surgical procedures, and achieving consistency between operators—make oversight impossible. This approach maintains surgeons’ independence, expedites innovation, and mitigates biases held by the surgical profession. Emergent cases and unexpected complications may necessitate innovation at a moment’s notice, which is amenable for this approach. However, it amplifies the effects of a surgeon’s own biases and conflicts of interest. This approach presumes rigorous ethical training, which is presently not met by current medical training or continuing medical education [5].

### Departmental and institutional oversight

Discussion with colleagues through informal conversation, approval by the chair, or case conferences provide departmental forms of regulation. The results of a policy including department chair approval and outcomes tracking for innovations have been reported at The Hospital for Sick Children with many surgeon-innovators commending its ease of use and noting that it encouraged them to innovate [6]. The benefit of departmental regulation includes rapid introduction of the innovation and preserved independence for the surgeon, who knows the patient’s anatomy the best. This approach does not mitigate the surgeon’s or institution’s potential conflicts of interest, and the degree to which pertinent ethical issues are considered likely varies widely by surgeon and institution.

Institutional ethics committees (IECs) that meet regularly to discuss anticipated alteration of procedures provide increasingly formalized oversight. The standards, scope, and role of such committees differ widely by institution, and no hospitals currently integrate them into routine surgical practice. IECs may contain bioethicists and lawyers among other professionals to provide multidisciplinary consultation. They may serve in a consultant role such that the decision-making rests with the surgeon or in a regulatory role where its decision may supersede that of the surgeon. These committees have played larger historic roles in medical, rather than surgical, decision-making in part because surgeons believe that ethical consultants may not truly understand surgical problems [7]. Advantages of this approach are its inclusion of multidisciplinary opinions, the possibility to teach peers, and the systematized consideration of pertinent ethical considerations. Challenges to the IEC method include differing standards between institutions, a slowed pace of innovation, and decision-making by professionals not directly involved in a patient’s care.

### Centralized oversight

Oversight boards organized by regional or national professional societies would provide the most centralized and standardized oversight for innovation. However, no surgical societies currently provide oversight committees for individuals who seek ethical support for an attempt at innovation. These committees would have the expertise to create committees to offer methodologically consistent and rigorous oversight for individual attempts at innovation. Such committees are currently hypothetical within the surgical community, but similar ones exist in medicine: the American Medical Association’s Council on Ethical and Judicial Affairs and other specialty societies have judicial and advisory responsibilities over certain ethics-related decisions. This centralized process would minimize individual bias and adds multidisciplinary knowledge, but may be slow and costly. Furthermore, it may be subjected to bias formed by the culture of current practice. Finally, these committees would consist of members not directly involved with the patient and may not appreciate the uniqueness of the case or patient’s anatomy.

### Formal research protocols

Some operative innovations have been tested in a research setting through clinical trials. Research is conducted with clinical equipoise and appropriate blinding and randomization to generate knowledge for a specific group of patients and requires formal research protocols with IRB oversight. Traditionally, the strongest evidence is provided by randomized control trials, but given low accrual, interpatient anatomic variation, and difference in skills between surgeons, most procedures are evaluated by single-operator/single-institution case series. IECs and IRBs are both institutional entities, but differ in organization and role. IECs are multidisciplinary teams that can aid physicians and surgeons through ethical questions similar to how a subspecialty consulting team may provide daily input on a patient at the request of the primary care team. IRBs are standardized committees that oversee formal investigative research and monitor ethics as well as efficacy. They are nationally mandated and standardized bodies designed to evaluate and oversee all formal research protocols. Their benefits include the multidisciplinary knowledge, minimization of conflict of interest, and nationally standardized implementation of research protocols to ensure safety and autonomy for patients and maintain integrity and accountability in research [8]. Their downsides include relatively slower review, which limits feasibility for emergent cases; significant costs; and oversight by evaluators who are removed from the clinical management of the patient [9].

## Ethical justification for formal oversight

The goal of oversight should be to provide practical structures that address ethical considerations delineated in earlier work: scientific validity, risk–benefit ratio, informed consent, protection of vulnerable populations, justice, and conflicts of interest [3, 10]. Scientific validity and risk–benefit ratio are “scientific factors” since both involve scientific and statistical estimations based on available objective research and expertise. Informed consent, protection of vulnerable populations, justice, and conflict of interest are considered “human factors” because they deal the less tangible subjective areas of interpersonal communication, social justice, and personal biases. The practical justification for this division is that scientific factors are best judged by colleagues in the same field who are familiar and experienced with the relevant pathology and anatomy. Human factors, on the other hand, benefit from a more multidisciplinary approach that recognizes the legal and cultural contexts behind these ethical principles. These have been expounded in the previous literature and are briefly summarized to motivate discussion for novel oversight mechanisms [11].

### Scientific factors

The scientific validity of an innovation depends on evidence of its safety and efficacy. Randomized control trials and meta-analyses are the gold standard in evaluating the clinical efficacy of an innovation, but the challenges of blinding and randomizing in surgery make conducting these trials difficult. Indeed, the prevalence and quality of RCTs in surgery remain low [12, 13].

Defining the risk–benefit ratio prior to any attempt at innovation is crucial. Surgical procedures may trade function to restore another function, decrease pain, or extend survival. Thus, precisely defining each patient’s values is crucial to align the goals of operative innovation with a patient’s own goals. Innovation carries a “learning curve” to reach maximal efficacy, and immediate risks may not be apparent and may depend on each patient’s anatomy [14]. Long-term risks of operative innovations may be difficult and take years of follow-up to quantify. Novel procedures bring financial burden, and ill-planned innovations risk harming the public reputation of the surgical profession [15].

### Human factors

Informed consent standards mandate that it is the responsibility of the surgeon to ensure that the patient understands the pertinent information necessary to make a choice about whether to proceed with a procedure. The information crucial to informed consent should include the innovative nature of the procedure, evidence to support it, and the surgeon’s experience with it [11].

Examples of vulnerable patients include unconscious patients, patients in emergency conditions, patients with refractory disease, and children, prisoners, ethnic minorities, socially marginalized persons, etc. [11]. Care should be taken to avoid tendencies, including implicit rationing that excludes certain patients, which may exploit vulnerable patients [16, 17].

Justice within innovation mandates that its risks and benefits are shared equally by society, including all geographic and socioeconomic groups. However, innovation may gravitate toward practices with a culture that encourages innovation and areas with minimal regulation of innovation. Innovative surgeons may attract attention from “in-the-know” patients connected to the medical community. Furthermore, early innovations not covered by insurers may limit representation by patients of lower socioeconomic status.

Conflicts of interest can be divided into financial and non-financial conflicts. Financial conflicts of interest occur when certain devices or surgical tools are preferred due to industry financial incentives. These conflicts are nationally monitored to an extent—the Sunshine Act in the USA requires that all payments from the industry to physicians are registered and open to the public, though does not mandate that physicians report these to their patients [18]. The achievement of innovation may also come with academic prestige or may be required to continue thriving in competitive fields of research for physicians or institutions.

## Oversight as quality improvement

**Fig. 1:**
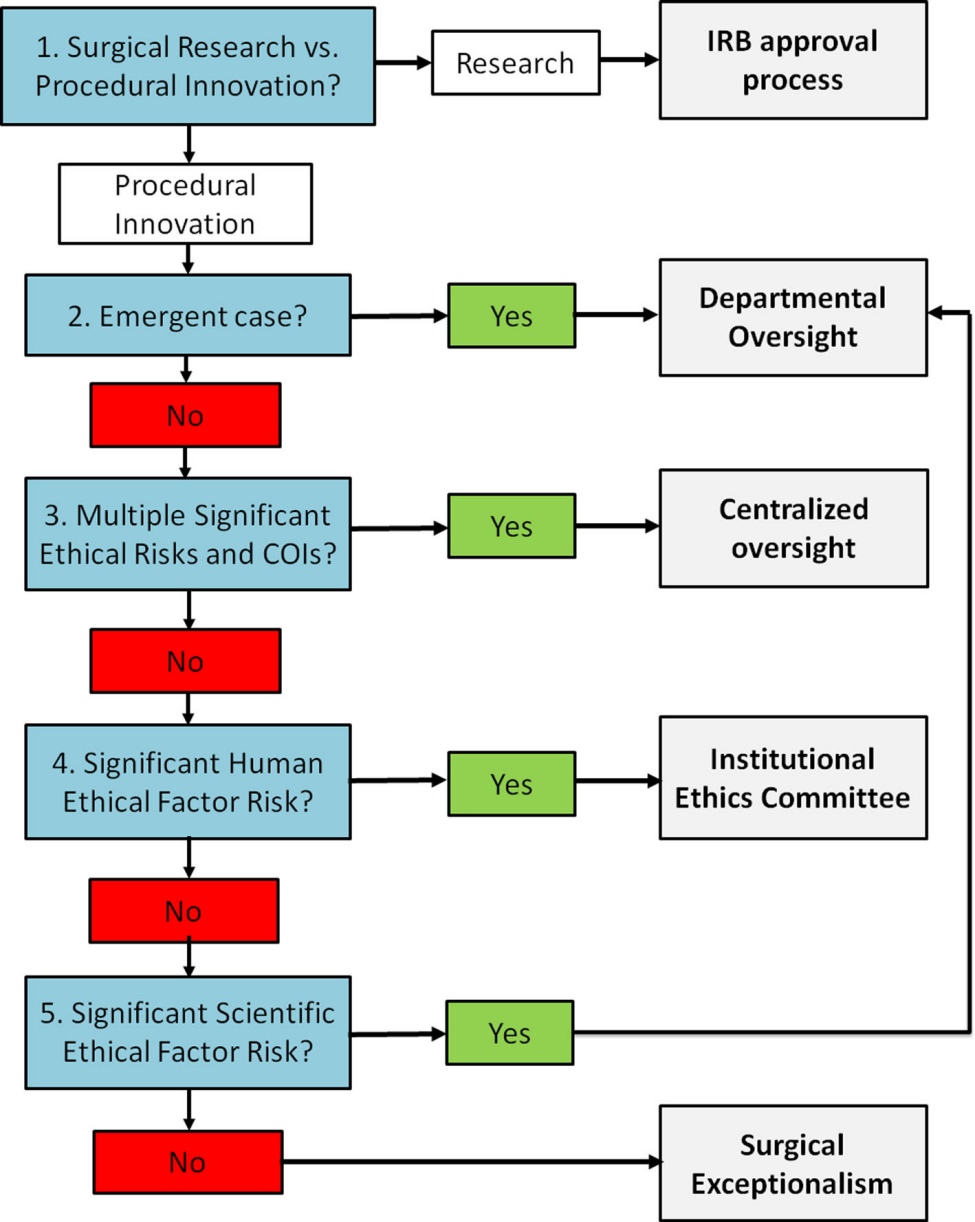
Framework for the determination of appropriate level of oversight

Standardized oversight structures can aid in mitigating ethical risks while protecting surgical independence in a quality improvement (QI) structure that shifts cultural practice rather than targets individuals. An ideal oversight framework would serve to accelerate innovation by protecting surgeons who were formerly too apprehensive about ethical and legal risks to innovate while not significantly slowing current surgeon-innovators. We propose a systematic, quality improvement framework to aid surgeons in the ethical introduction of surgical innovations (Fig. 1). This framework builds on The Society of University Surgeons Surgical Innovations Project Team’s position statement by stratifying different levels of innovation [19]. Surgeons could utilize existing tools to identify an innovation as such and then apply this framework to determine the appropriate level of oversight [19, 20]. This approach would maintain surgical independence and dignity and encourage the surgeon to take ownership in the ethical care of their patient. In general, operative innovations that present greater ethical challenges should warrant increased oversight. Other factors to weigh include the experience of the surgeon and the emergence of the case.

This framework should be adopted in a QI mechanism with measurable outcomes. QI requires transparency; rigorous data collection and analysis; and openness to adjust. Relevant outcomes include surgeons’ sense of support supported while innovating, the usability of this framework, and patients’ understanding of an innovation. Objective measures include number of innovations performed annually and lawsuits from adverse outcomes or miscommunication. Standardized data collection on the administrative aspects prior to an innovation (i.e., ease of committee meeting, adequate time for a department to deliberate an innovation, etc.) could generate valuable information on how to implement this oversight framework efficiently. Prospective data capture from surgical innovations themselves could provide a wealth of information to other surgeons considering similar procedures and may facilitate collaboration as well as study of an innovation. The mindset of a learning QI system should continually incorporate data analysis to improve the framework’s content and delivery. Voluntary, surgeon-led QI initiatives depend on mutual trust and have demonstrated success in other elements of surgical care [21].

An important initial delineation for this framework is distinguishing research and individual clinical contexts. The distinction of these rests on their respective motivation: the primary goals of operative innovation in the clinical and research contexts, respectively, are beneficence to optimize patient care and experimental evaluation to generate generalizable knowledge. Experimental techniques intended to test the new technique with equipoise fall into the research category that receives oversight from IRBs. An example is single-port laparoscopic cholecystectomy for porcelain gallbladder with considerable malignant potential. Traditional laparoscopy already carries an acceptable risk for this pathology, and this single-port approach is not an innovation for an individual patient’s unique anatomic or pathologic circumstances, but rather as a challenge to multiport laparoscopy.

An operative innovation may at the same time be experimental and introduced by the surgeon specifically for a patient thought to derive benefit from it; these cases fall into the innovation for individualized clinical benefit category. Oversight in this category includes surgical exceptionalism, informal discussion with colleagues, formal departmental conferences, IECs, and regional/national ethics committees (Table 1). The ethical factors that determine the appropriate level of oversight include the aforementioned scientific factors and human factors. Practical considerations unique to surgery such as expertise of the surgeon and emergence of the case also factor into this determination. Illustrative cases are described in Table 2, though as a caveat, no consensus about what constitutes surgical innovation exists and individuals may vary in scenarios they consider innovation.^1^

**Table 2:** Illustrative case examples of surgical innovations appropriate for different oversight levels

Oversight level	Example for non-emergent cases	Ethical risks
Surgical exceptionalism	Modification of port location to facilitate laparoscopic cholecystectomy in an adult patient with situs inversus totalis who is able to provide informed consent	No significant risks
Departmental	Approach and location of renal transplantation in renal failure patient with extensive retroperitoneal scarring from previously irradiated sarcoma	Scientific risks
IEC^a^	Novel combined open/neuroendoscopic approach for a unusual arteriovenous malformation in an obtunded patient without a known advance directive	Human risks (informed consent)
Regional/national	Transvaginal lysis of peritoneal adhesions for a patient wishing to avoid visible scars by a program with financial ties to transvaginal endoscope manufacturer	Scientific risks, financial conflict of interest
IRB^b^	Thoracoscopy versus thoracotomy for pulmonary lobectomy in severe COPD patient	–

^a^Institutional ethics committee

^b^Institutional review board

The ideal cases for surgical exceptionalism are limited to those in which the presence of any regulation at all is unnecessary or overly burdensome. Such procedures without significant ethical challenges involving efficacy or decision-making will not require further oversight. Relevant caveats to this approach are that surgeon discretion presumes training in identifying innovation and in surgical ethics and that only innovations that do not significantly depart from standard of care warrant no additional oversight since the risk–benefit ratio is not as predictable in innovations that depart from standard. Further, the innovation should be discussed with other members of the surgical and postoperative care teams, including anesthesiologists, critical care physicians, and nursing staff so they can provide input and also anticipate changes required in their care. An example case for surgical exceptionalism is the utilization of a new port location to facilitate laparoscopic cholecystectomy in an adult patient with situs inversus totalis who is able to provide informed consent.

Cases that involve challenges to scientific ethical factors, but not human ethical factors, may benefit from departmental oversight. These innovations may be supported by lower quality preclinical evidence or have poorly defined risk–benefit ratios, but there are no risks in the communication between the surgeon and the patient and no conflicts of interest for the surgeon. The surgeon’s own colleagues would be best poised to refine the innovation to maximize benefits to the patient, but as the surgeon knows the patient’s anatomy and clinical history the best, the decision to innovate remains with the surgeon and patient. Surgeons with extensive experience with the anatomic features involved in a proposed innovation may be well prepared to undertake an attempt at innovation without oversight by colleagues as their expertise provides them with the best possible assessment of efficacy and safety. Like in surgical exceptionalism, anesthesiologists and postoperative teams should be included. The multidisciplinary knowledge of IECs and centralized oversight committees, which could aid in communicating informed consent or assessing patient vulnerability, are unnecessary since no human ethical factors are challenged. Under this framework, departmental discussion would be appropriate in determining the approach and optimal extent of resection for a large complex skull base lesion that invades nearby neurovascular structures and is expected to be difficult to remove due to prior irradiation.

Innovations that involve challenges to human ethical factors (with or without scientific ethical factors) step up to oversight by IECs. IECs benefit from a diverse range of opinions due to their multidisciplinary nature and are consequently poised well to manage situations presenting complex ethical challenges. Multidisciplinary institutional committees containing ethicists and lawyers have the expertise to help surgeon-innovators navigate difficult informed consents, ensure the protection of this vulnerable patient, and mitigate conflicts of interest. One weakness of this framework is that IECs differ in role, scope, and make-up by institution. Collaboration by surgical and ethical societies to standardize or create minimal requirements for IECs is necessary to ensure these committees are equally prepared to assess this level of surgical innovation. Major academic hospitals may partner with non-academic centers to ensure their access to IEC expertise. As a caveat, emergent cases that a surgeon deems to warrant an operative innovation may supersede other ethical considerations due to time constraints, so an emergent innovation may warrant a lower level of oversight. For example, a surgeon managing an adolescent with cystic fibrosis complicated by bronchiectasis who presents with penetration multiple gunshot wounds to the chest may seek a modified conservative approach for repair to maximize salvage of lung parenchyma, but the patient’s condition may demand action before an IEC can convene. The surgeon must depend on more expedient forms of oversight such as discussion with colleagues or post hoc case conferences in these emergent settings.

A more centralized oversight process coordinated by regional or national professional societies is warranted to ensure the ethical introduction of operative innovations that involve an institutional conflict of interest, such as holding financial stakes in a company funding an innovation, in addition to human or scientific ethical challenges. While the members of these centralized committees would have similar multidisciplinary expertise as IECs, they mitigate the effects of institutional conflicts of interest. Centralized committees are entirely hypothetical in surgery, and a major barrier to formation is restructuring professional societies to incorporate them. Patient advocacy organizations could work with state and national governments to help fund these committees. An example case is alveolar bone graft prior to odontic maturation for cleft palate repair in a child flown in pro bono from an underdeveloped country with the expectation of the department using this case to promote its humanitarian work. It may be reasonable to innovate on this patient early given that the patient may not have future access to medical care; however, there are human risks (justice for patient with less access to care, vulnerable child patient) and scientific risks (very novel procedure, so unclear risk–benefits) at play, as well as the department’s benefiting from advertising this humanitarian procedure. Once formed, centralized oversight committees may integrate with IECs by sending unbiased representatives to consult with them to maintain institutional independence and to accelerate decision-making for time-dependent procedures. Again, emergent procedural innovations that would otherwise warrant such oversight may depend on less oversight given time restraints.

Current challenges requiring further exploration include tools for surgeons to identify innovation and conflicts of interest, the development of standardized case conferences and IECs, and infrastructure that integrates oversight seamlessly with surgical care. Data collection on the efficiency and ease of the framework would aid procedures in effective implementation of the framework. Ethical considerations may be complex and surgeon-innovators may seek multiple types of oversight simultaneously. For instance, IRBs do not often contain multiple surgical subspecialists as reviewer, so an IRB-approved study may additionally benefit from departmental oversight of risk–benefit calculations. Multi-institutional IRB-approved studies may similarly benefit from departmental or regional oversight to help weigh these calculations. Different departments may be especially well attuned to the different conflicts of interest and levels of ethical training in their group, which could aid IRBs. IRBs may benefit from inclusion of subspecialist consultants as well. The role of insurers who decide which innovations to cover is important to also consider as they influence which patients receive innovations. The role of insurers in this framework may vary depending on the healthcare system; for example, a government-run single payer system acts broadly in citizens’ interests, so it may conduct process checks for adherence to this framework as a requirement for coverage of innovations.

The ultimate decision on whether to seek oversight currently rests with surgeons. This proposed framework does not reduce a surgeon’s independence and ownership over their patients; rather, it aims to protect patients from risk and support surgeons through ethical quandaries to allow them to keep their focus on innovating in the operating room. Previous experience even suggests some regulation may actively promote a culture of innovation through offering assurance and confidence to innovators that they are innovating in an approved ethical manner [6]. This quality improvement framework builds on the pillars of surgical professionalism and education: competence, integrity, humility, and consistency. This framework seeks to align with historic surgical ethos to create a culture of continual self-improvement in a learning environment wherein everyone from patients to surgical interns to renown surgeon-innovators benefits. These proposed levels of oversight provide a consistent and ethically sound method to introduce new innovations. The framework should be introduced with care to ensure all faculties understand its purpose and understand how to use it. It should also accommodate local regulation and oversight, the specific subspecialties in a hospital, and the patient populations’ needs. Continuous improvement and adjustments of the framework are necessary to ensure potential benefit to patients.

## Conclusion

Current methods to address ethical challenges to operative innovation are inconsistent and open surgeons and patients to risk. Possible oversight mechanisms for operative innovation range from no oversight to formal IRB review. Certain oversight mechanisms may be well suited to regulate an attempt at innovation depending on the type and degree of pertinent ethical challenges to ensure the continued advancement of the field while protecting patients and supporting surgeons.
